# The Influence of Fibre and Fly Ash Additions on the Properties of Self-Compacting Concrete

**DOI:** 10.3390/ma18112565

**Published:** 2025-05-30

**Authors:** Gabriela Rutkowska, Jacek Szulej, Paweł Ogrodnik

**Affiliations:** 1 Institute of Civil Engineering, Warsaw University of Life Sciences—SGGW, Nowoursynowska 166, 02-787 Warsaw, Poland; gabriela_rutkowska@sggw.pl; 2 Faculty of Civil Engineering and Architecture, Lublin University of Technology, 20-618 Lublin, Poland

**Keywords:** self-compacting concrete, polypropylene, glass, steel fibres, compressive strength, flexural, CO_2_ emissions

## Abstract

Self-compacting concrete (SCC) is an innovative building material that is distinguished by its ability to flow and fill forms without the need for mechanical vibration. The aim of this research was to determine the effect of different types of fibres—steel, glass, and polypropylene—on the properties of both the fresh mix (consistency, density, air content, and viscosity) and the hardened concrete (compressive strength, tensile strength in bending, density, water absorption, and frost resistance). Attention was also paid to CO_2_ emissions associated with cement production and the potential of their reduction by using alternative materials. The results of the conducted research demonstrate that, in terms of enhancing the mechanical properties of self-compacting concrete (SCC), the incorporation of glass fibres (GFs) leads to the most significant improvements in compressive and flexural strength—by 1.6% and 29.2%, respectively. Therefore, these fibres can be recommended for use in high-performance structural applications, such as precast elements, load-bearing components, and structures subjected to dynamic loading. Polypropylene fibres (PPFs), owing to their ability to reduce water absorption by 7.3%, may be suitable for elements exposed to high humidity and shrinkage risk, such as tunnels, fire-resistant barriers, or insulating layers. Steel fibres (SFs), in turn, have proven particularly effective in SCC used for industrial flooring and other elements exposed to cyclic dynamic loads.

## 1. Introduction

Concrete is currently the most commonly used composite material among those produced by man, and the second-most used after water in the entire complex of materials used. It is a key construction material, made from local raw materials—aggregate, cement, and water—and enriched with innovative additives and admixtures (including: ash from sewage sludge incineration, natural mineral zeolite, recycled ceramic aggregate, and waste crushed glass), whose production and application technology are constantly developing, resulting in the creation of new recipes [1,2,3,4,5,6,7,8]. The European Union is introducing increasingly rigorous standards and regulations that require innovative solutions in the design, construction, and use of buildings to meet the requirements of sustainable development. One of the main problems in construction is the high CO_2_ emissions resulting from the production of cement, which is the primary component of concrete [9,10,11]. The International Panel on Climate Change (IPCC) in its sixth report [12] states that in 2023, compared to 1980, annual carbon dioxide emissions from human activity have doubled to 41 gigatonnes. The construction sector is one of the key areas responsible for greenhouse gas emissions, both through the production processes of building materials and the operation of buildings. Currently, the global production of Portland cement is about 4.6 billion tons [13], which contributes to about 8% of global CO_2_ emissions [14]. It is estimated that each ton of cement produced generates 0.6 to 0.8 tons of CO_2_, making this industry one of the world’s leading greenhouse gas emitters [15]. The European Union, as part of the “Fit for 55” initiative, aims to reduce CO_2_ emissions by 55% by 2030 compared to 1990 levels. Currently, concrete production does not meet future emission standards, which necessitates action to reduce them. Key strategies include [16,17]:

- Increasing energy efficiency—modernisation of cement plants and the use of new technologies, including alternative fuels, e.g., biomass, can significantly reduce CO_2_ emissions. According to the European Cement Manufacturers Association (CEMBUREAU), such actions can reduce emissions by up to 30% [17].
- Reducing the share of clinker in cement—clinker is the most carbon-intensive part of cement. Replacing it with supplementary materials, such as fly ash or blast furnace slag, can reduce CO_2_ emissions by up to 40%, while also enhancing the mechanical properties of concrete [18].
- Using CCS (Carbon Capture and Storage) technology—capturing and storing CO_2_ directly from the production process has the potential to reduce emissions by up to 85–95%. However, implementing this technology requires substantial investment and international cooperation [18].

**Figure 1:**
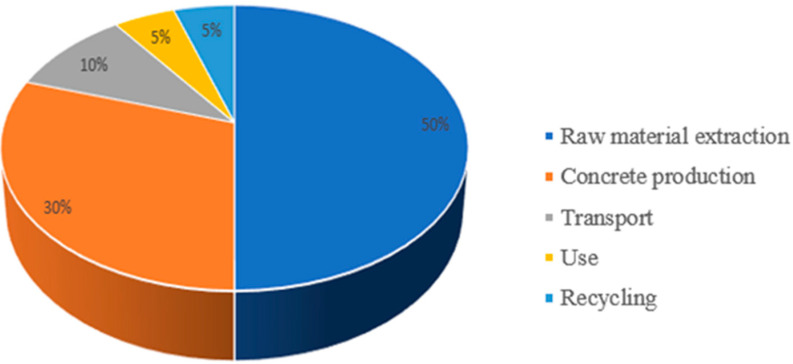
CO_2_ emissions during the life cycle of concrete.

For CCS to play a key role in reducing emissions, further research, the development of innovative solutions, and cooperation between governments and the private sector in its commercialisation are essential. Appropriate political and regulatory support is also necessary to achieve ambitious CO_2_ emission reduction targets, especially in the cement sector, which remains one of the largest emitters of greenhouse gases. Achieving these goals requires decisive political action, such as implementing financial support schemes, tax breaks, and other incentives for companies investing in low-emission technologies. As indicated by CEMBUREAU [17], “accelerating the implementation of low-emission technologies depends on effective political and regulatory support, including financial mechanisms that encourage companies to invest in sustainable solutions”. Similar conclusions can be drawn from the research published in the journal “Energy Policy”, according to which “appropriate regulations and support policies can create favourable conditions for industries to effectively reduce CO_2_ emissions. Examples of such actions include emission trading schemes and grants for research and development of low-emission technologies”. Thanks to such initiatives, the cement sector has a chance to meet climate goals, including a 55% emissions reduction by 2030, in line with the EU’s “Fit for 55” strategy. The introduction of appropriate policies will not only contribute to reducing emissions, but also enhance the competitiveness of the European industry in the global market, while supporting innovation and sustainable development. Figure 1 shows CO_2_ emissions during the life cycle of concrete [16,17].

Therefore, in addition to the previously mentioned additives and admixtures that partially replace cement and aggregate, it is becoming so important to pursue new technologies and innovative ingredients that can reduce the carbon footprint of concrete while maintaining its high strength and functionality. In this context, an important solution is self-compacting concrete (SCC), which has gained significant popularity in recent decades. SCC, developed in Japan in the late 1980s, facilitates the construction of structures with complex shapes and dense reinforcement, while improving working conditions on the construction site. A key aspect of innovative concrete mixtures is their composition, enriched with additives such as polypropylene, steel, or glass fibres, which enhance the strength, durability, and frost resistance of concrete [19,20,21,22,23]. According to EN 206+A2:2021-08, self-compacting concrete is a material that flows and compacts automatically, filling the formwork along with the reinforcement and other structural elements [24]. It is more demanding in production than traditional concrete, as it is necessary to ensure the appropriate rheological properties of the mixture and maintain them until it is placed. The stability of the SCC mixture is crucial—if it is low, the concrete may lose its properties. In terms of component storage, SCC is no different from traditional concrete. However, it is crucial to monitor the moisture content of the fine aggregate and the grain size of the cement and mineral additives, because even small changes in the water content can affect the properties of the mixture. SCC mixtures are characterised by high fluidity, which is associated with the risk of significant pressure on the formwork. In order to prevent leaks, it is necessary to use tight joints and closed forms. SCC is transported in trucks with a mixer, because any remaining mixture can quickly stiffen and lose its ability to flow. To prevent this, the mixture should be mixed during transport at low speed to avoid segregation of the components. SCC can be laid using various methods, including concrete pumps; however, in the case of special mixtures, e.g., with the addition of fibres, it is necessary to carry out laying tests to confirm their suitability. Self-compacting concrete is an innovative solution in construction that significantly facilitates the implementation of complex structures. However, its use requires specialist knowledge and strict technological control to ensure optimal properties and durability of the finished element [21].

Self-compacting concrete significantly improves the quality of construction, especially in the case of elements with complex shapes and dense reinforcement. Its use improves working conditions, although at the same time it requires specialist knowledge and precise technological control. In Poland, SCC constitutes about 2% of the total production of ready-mix concrete, which results from the difficulties in its design and execution. Among the additives to SCC, polypropylene fibres stand out, which reduce the risk of cracking in the initial period of cement hydration. They are also expected to improve the durability of concrete and reduce water absorption and permeability [22]. In the paper [24,25], SCSFRC (steel fibre-reinforced self-compacting concrete) mixtures were designed, prepared, and tested to investigate a wide range of rheological and mechanical conditions. This allowed for the identification of the optimal proportions of the constituent materials. The research is ongoing on the effect of fibres on the frost resistance of SCC—the results of Persson [26] indicate that after 300 freeze–thaw cycles, self-compacting concrete with a small amount of polypropylene fibres did not demonstrate frost resistance. However, according to the research by Szwabowski and Miera [27], effective frost resistance of SCC requires the use of an air-entraining admixture. Comparisons have shown that the fibres themselves can worsen the workability and flow of the mixture, and their effect depends on the w/c ratio. In turn, steel fibres, often used in SCC, improve its compressive and tensile strength, reducing the formation of cracks [28,29,30,31]. Ponikiewski’s research shows that although steel fibres slow down the flow of the mixture and reduce its spread diameter, they also increase its strength [32]. Experiments have shown that the greater the amount of steel fibres, the better the compressive strength. Glass fibres, on the other hand, allow for tight filling of formwork without the need for vibration, improving durability and preventing sedimentation. The addition of these fibres increases scratch resistance and improves bending and cracking strength. Studies have shown that the effectiveness of fibres depends on their quantity, length, thickness, and distribution in the mixture. Experiments have shown that SCC with the addition of glass fibres is characterised by better plasticity and compressive and tensile strength, reduced water absorption, and increased durability of concrete [33,34,35,36]. In the paper [37], waste glass fibres were added to self-compacting concrete. An increase in the compressive, tensile, and bending strength of the samples and an increase in the Young’s modulus values were obtained. The authors of the paper [38] estimated the most optimal share of propylene fibres in the concrete mix due to the greatest increase in compressive and tensile strength. They obtained a result of 2%. In the scientific research [39], the authors investigated the mechanical properties of self-compacting lightweight concrete (SCLC) made of lime powder and reinforced with polypropylene fibres. The results of the research show that adding fibres does not affect the compressive strength, but slightly improves the modulus of elasticity and tensile strength. The aim of the SCC research was to determine the effect of different types of fibres—steel, glass, and polypropylene—on the properties of both the fresh mix (consistency, density, air content, and viscosity) and hardened concrete (compressive strength, tensile strength in bending, density, water absorption, and frost resistance). This study also focused on CO_2_ emissions associated with cement production and the potential for their reduction through the use of alternative materials.

## 2. Materials and Methods

The tests were carried out in accordance with procedures based on the guidelines outlined in the applicable regulations and EU standards. In order to determine the effect of polypropylene, glass, and steel fibres on the selected properties of the concrete mix and mature concrete, samples of self-compacting concrete SCC of class C30/37 with consistency S3, exposure class XC3 in accordance with EN 206+A2:2021-08 [24], were made. The formulation was developed using an empirical and experimental method commonly applied in the industry, with the support of specialised computer software owned by a company with over 100 years of tradition in the production of cement, ready-mix concrete, and aggregates. Portland ash cement, CEM II/A-V 42.5 R, from the Chełm cement plant was used to prepare the mix, which met the requirements of the EN 197-1:2012 standard—Table 1 [40].

The same granulometric composition of the aggregate fraction (0–16 mm) selected using the limit curve method was assumed in all samples. The bulk density of the coarse and fine aggregate was determined by the pycnometric method according to EN 1097-6:2002 [42]. The density of sand and gravel was the same and amounted to 2.63 g/cm^3^. Tap water compliant with EN 1008:2004 [43] and fly ash with a density of 2.14 g/cm^3^, coming from the Enea power plant produced in Świerże Górne and the admixtures Isoplast 1528 and Isoflow 7495, were used for laboratory tests. The properties of the admixtures are presented in Table 2.

**Figure 2:**
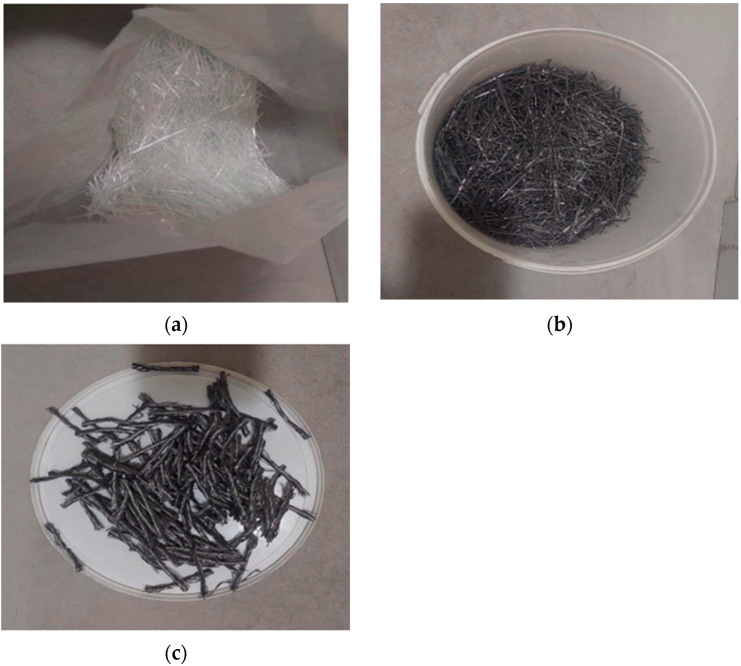
Types of fibres used for testing: (**a**) glass fibres HP 67/36, (**b**) steel fibres STALMIX 50, and (**c**) polypropylene fibres.

To strengthen the structure of concrete, polypropylene, glass, and steel fibres were used. Figure 2 shows the fibres.

The following types of concrete mixtures were prepared for laboratory tests:

- BZ—reference concrete without added fibres;
- WPO—concrete with added polypropylene fibres;
- WSZ—concrete with added glass fibres;
- WST—concrete with added steel fibres.

Table 3 presents the composition of the individual concrete mixtures. The same amount of fly ash was used for each mixture in the amount of 150 kg/m^3^.

**Figure 3:**
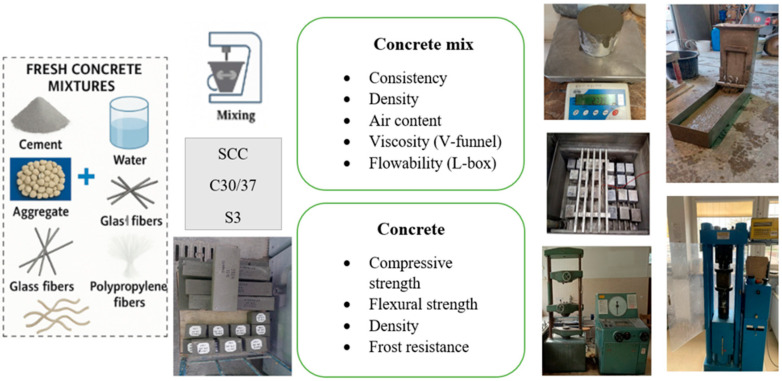
Diagram of the experimental research program.

Figure 3 shows a block diagram of the experimental research program.

The following tests were carried out on fresh concrete mixes:

- Consistency, according to EN 12350-2:2019-08, using the flow table method [46];
- Density, according to EN 12350-6:2019-08 [47];
- Air content, according to EN 12350-7:2019-08, using the pressure method [48];
- Viscosity using a V-funnel according to EN 12350-9:2012 [49];
- Flowability using an L-box according to EN 12350-10:2012 [50].

The following concrete tests were carried out:

- Compressive strength, according to EN 12390-3:2019-07, on samples of dimensions 10 × 10 × 10 cm (after 3 samples) [51];
- Flexural strength according to EN on samples with dimensions of 10 × 10 × 40 cm (3 samples each) PN-EN 12390-5 [52];
- Density, according to EN 12390-7:2019-08 on samples with dimensions of 10 × 10 × 10 cm (3 samples each) [53];
- Frost resistance, according to PN-88/B-06250 on samples with dimensions of 10 × 10 × 10 cm (12 samples each) [54];
- Water absorption according to PN-88/B-06250 on samples with dimensions of 10 × 10 × 10 cm (3 samples each) [54].

The tests were carried out in the Construction Laboratory at the Faculty of Civil Engineering, in the Physical Processes Laboratory at the Water Center of the Warsaw University of Life Sciences. The compressive strength fc test was performed after 28 days of curing in a hydraulic testing machine H011 Matest (Bergamo, Italy), flexural strength using a hydraulic press—ZD40 (Distrelec, Bremen, Germany), and frost resistance in the chamber of the Toropol (Opole, Poland). Based on the obtained average strengths, the result was converted into cubic samples with a side of 15 cm and the concrete class was determined. The frost resistance test was performed on samples with dimensions after 28 days of concrete curing for 150 freeze–thaw cycles.

## 3. Test Results and Their Analysis

### 3.1. Concrete Mix

Table 4 presents the test results of individual concrete mixes.

**Figure 4:**
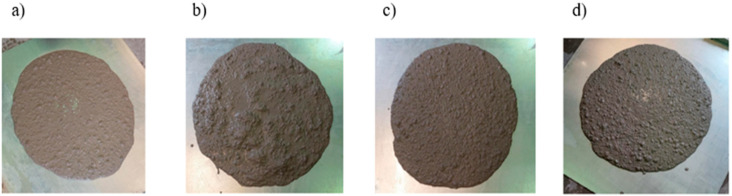
Consistency of (**a**) BZ, (**b**) WPO, (**c**) WSZ, and (**d**) WST.

All mixtures have a similar consistency in the range of 595–625 mm, marked as SF1, indicating mixtures with good workability (Figure 4). The reference concrete mix BZ achieved a flow of 625 mm, which is the reference point in the analysis. WPO (595 mm)—the addition of polypropylene fibres caused a reduction in flow by 30 mm, which may result from their tendency to increase the viscosity of the mixture and retain water. WSZ (600 mm)—similarly to WPO, the inclusion of glass fibres reduced flow, but to a lesser extent. WST (620 mm)—steel fibres caused a minimal reduction in flow by 5 mm, which suggests that they limit the flowability of the mixture less than synthetic fibres. Based on the obtained results, it was found that polypropylene and glass fibres significantly reduce the fluidity of the mixture compared to BZ, while steel fibres have little effect on it. Polypropylene (PP) fibres have the greatest impact on fluidity, which can hinder self-compaction. Glass (PP) and steel (PP) fibres maintain good fluidity, making them more suitable for SCC. All mixtures have similar densities, ranging from 2.292 to 2.314 kg/m^3^. PPP has the highest density (2.314 kg/m^3^), which may suggest fewer air voids or a higher solids content. PPP has the lowest density (2.292 kg/m^3^), but the differences are minimal. PP fibres have a very low density, which may explain the lower value, while steel fibres, as a high-density material, increased the unit mass of the mixture, which may positively affect the strength of the mature concrete. For SCC, adequate stability is essential to avoid the segregation of the components. PPP (PP) improves the stability of SCC due to its higher density. WPO (polypropylene fibres) can increase the risk of segregation, which is unfavourable for SCC. The addition of fibres did not affect the amount of air in the mix; all mixes have the same air content—3.0%, which means that their porosity and potential resistance to freezing are comparable. BZ and WSZ mixes have the lowest viscosity (6.5 s/VF1), which means that they are more fluid. Polypropylene fibres significantly increased the viscosity (9.5 s/VF2), which may indicate a more viscous and slower flowing mix. WST has an intermediate value (7.0 s/VF1), which suggests moderate viscosity, so SCC can still self-compact well. Polypropylene fibres worsen the flowability, likely due to increased viscosity. This may be due to the higher viscosity. Glass fibres improve flowability over BZ, which means they can improve the mould-filling ability.

### 3.2. Mature Concrete

#### 3.2.1. Compressive Strength

**Figure 5:**
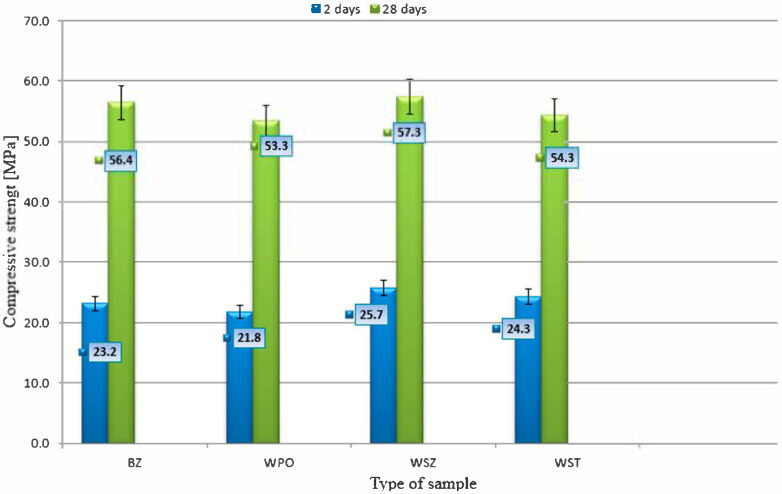
Compressive strength after 2 and 28 days of curing.

**Figure 6:**
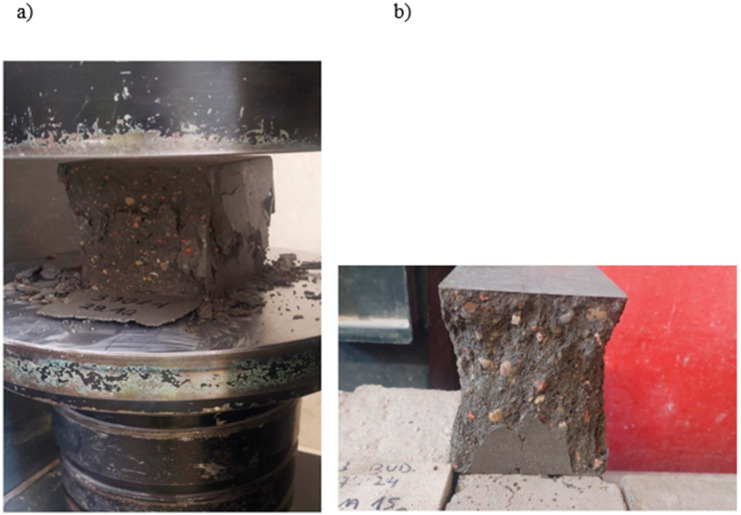
Compressive strength test: (**a**) view of the sample in the press and (**b**) view of the sample after the test.

Figure 5 shows the compressive strength results and Figure 6 the compressive strength test.

Based on the conducted tests, it was found that glass fibres (WSZs) increase strength both in the early period and after 28 days, making them the best choice in terms of compressive strength. Steel fibres (WSTs) improve early strength, but after 28 days the result is slightly lower than that of the reference concrete. Polypropylene fibres (WPOs) reduce the compressive strength in both periods. While they may offer other advantages (e.g., reduced shrinkage or improved fire resistance), they do not have a positive effect on compressive strength. The highest compressive strength of 57.3 MPa was observed for WSZ concrete samples, while the lowest, 53.3 MPa, was recorded for WPO concrete samples. In accordance with EN 206+A2:2021-08 [24], the compliance criterion for the obtained compressive strengths was checked using the specified formulas.

f_cm_
*≥* f_ck_ + 4 and f_ci_ ≥ f_ck_ − 4 (1)

where:

f_cm_—average compressive strength [MPa];

f_ck_—characteristic strength [MPa];

f_ci_—single result from a series of samples [MPa].

#### 3.2.2. Flexural Strength

**Figure 7:**
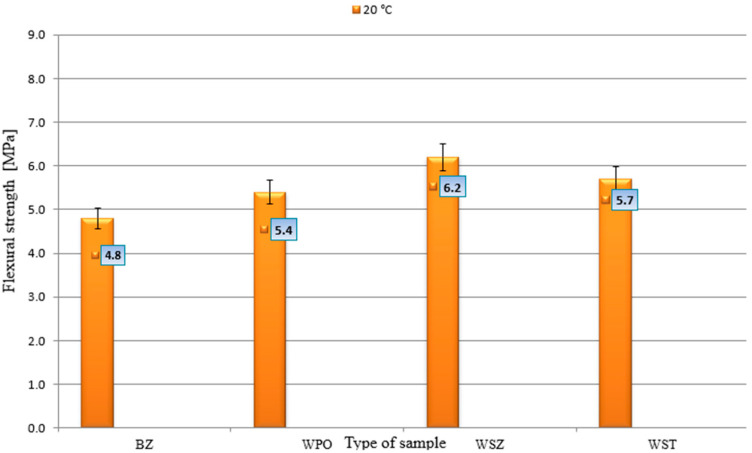
Flexural strength.

Figure 7 shows the flexural tensile strength results for concrete.

The lowest flexural strength, 4.8 MPa, was obtained by the reference concrete. The lack of fibres makes the concrete brittle and susceptible to cracking. Concrete with glass fibres achieved the best result of 6.2 MPa, representing a +29.2% increase compared to BZ. Glass fibres act as micro-reinforcement, improving resistance to cracking—ideal for applications requiring high resistance to bending. The addition of steel fibres increased strength by 18.8% and polypropylene fibres by 12.5%. Steel fibres absorb dynamic stresses well and improve impact strength, while the main advantage of WPO fibres is their ability to reduce shrinkage and micro-cracks. The introduction of fibres to self-compacting concrete (SCC) significantly affects its mechanical properties, especially in terms of compressive and bending strength. The best choice for SCC is glass fibres (WSZs), which provide the highest improvement in flexural strength (+29.2%) and slight increase in compressive strength (+1.6%). This makes them ideal for structures requiring fracture toughness and mechanical strength. Polypropylene fibres (WPOs) improve SCC resistance to micro-cracks and shrinkage, but at the same time reduce its compressive strength (−5.5%). They can be used in SCC in structures exposed to plastic shrinkage and extreme temperatures, e.g., in tunnels or fire protection elements. Steel fibres (WSTs) increase flexural strength (+18.8%), but may slightly reduce compressive strength (−3.7%) and impede the flowability of the SCC mixture. They are a good choice for industrial floors and structures subjected to high dynamic loads. The final choice of fibre type should take into account the specific design requirements and the necessary balance between strength, workability, and shrinkage resistance.

#### 3.2.3. Frost Resistance

Table 5 presents the results of concrete samples after the frost resistance test, and the drawing shows a view of the samples.

The frost resistance test of SCC after 150 freeze–thaw cycles allows for the assessment of the influence of different fibres on the durability of concrete in low-temperature conditions. The smallest decrease in strength was observed in SCC with glass fibres (WSZs) at (−35.00%) indicating the best resistance to freeze–thaw cycles. The greatest decrease in strength was noted for the reference concrete (BZ) (−43.53%), confirming that fibres significantly enhance frost resistance. Steel fibres (WSTs) and polypropylene (WPO) also improve frost resistance (−38.55% and −41.14%), but are less effective than WSZ. The smallest mass loss was noted for BZ (−0.242%), which suggests that concrete without fibres may have a more closed-pore structure. The highest mass loss was observed for SCC with polypropylene fibres (WPOs) (−1.443%), which may indicate greater porosity or susceptibility to surface fragment detachment. Glass fibres (WSZs) and steel fibres (WSTs) had moderate mass loss (−1.226% and −1.278%, respectively).

Three criteria were adopted to assess the degree of frost resistance, the fulfilment of which determines the degree of frost resistance achieved:

- No cracks in the samples after all freezing and thawing cycles;
- A difference of no more than 5% in the mass of samples soaked in water before and after the frost resistance test;
- A decrease in compressive strength between reference samples and frozen samples of no more than 20%.

**Figure 8:**
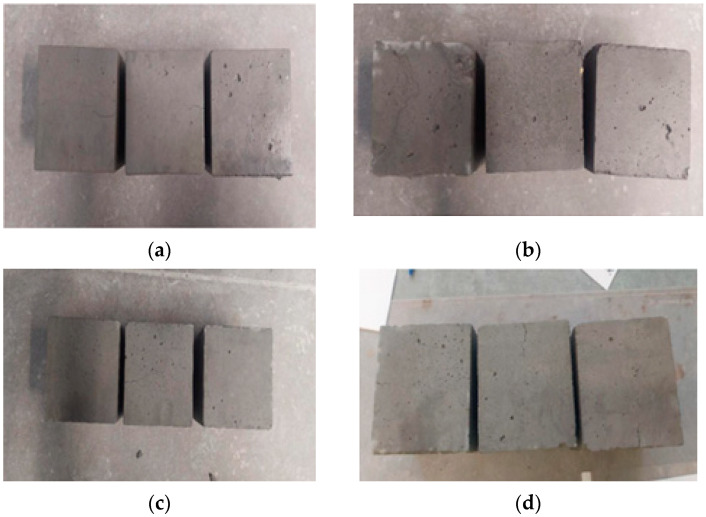
Concrete samples after frost resistance test: (**a**) BZ, (**b**) WPO, (**c**) WSZ, and (**d**) WST.

Based on the tests conducted, it was found that neither the reference concrete nor the concretes containing various types of fibres met the above criteria. Therefore, the tested concretes are not resistant to low temperatures. Figure 8 shows concrete samples after frost resistance testing.

#### 3.2.4. Water Absorption

The reference concrete (BZ) achieved a water absorption result of 6.62% and serves as a reference point for assessing the effect of fibres. The addition of polypropylene fibres resulted in a 7.3% decrease in water absorption, indicating lower porosity and better water resistance. These fibres improve resistance to shrinkage and micro-cracks, which has a positive effect on the durability of SCC. They do not significantly improve compressive strength, but can help reduce corrosion caused by water absorption. These fibres are a good choice for SCC in environments with high humidity and risk of shrinkage. The addition of glass fibres resulted in the greatest decrease in water absorption (−10.9%), which means the lowest porosity and the best durability. They improve resistance to cracking and bending (+29.2%), making them the best choice for structures exposed to high mechanical loads. They are most suitable for SCC with high strength and durability, such as in precast and structural elements. Steel fibres (8.38%) increased water absorption compared to the reference concrete, which may indicate greater porosity and risk of degradation under water and frost exposure. They significantly improve flexural strength (+18.8%), but their effect on compressive strength is minimal. They are suitable for SCC used in structures subjected to dynamic loads (e.g., industrial floors and pavements), but require additional protection against moisture.

#### 3.2.5. Concrete Density

Based on the conducted concrete tests, it was found that BZ, the reference concrete, obtained the lowest density (2.313 kg/m^3^), indicating that the introduction of fibres affected the increase in volumetric mass. WPO concrete showed a slight increase in density (2.354 kg/m^3^, +1.77%), the lowest among all fibre-reintroduced mixes. Polypropylene is a material with a very low density, so the change is not significant. A good choice when the low dead weight of SCC and improved resistance to micro-cracks and shrinkage are important. A slightly higher density than BZ, but lower (2.336 kg/m^3^, +0.99%) than WST and WPO was observed in concrete with glass fibres. It offers density and mechanical properties, including the best bending strength and lowest water absorption. This makes it the best choice for SCC, where high durability and strength are key. The highest density was obtained in concrete with the use of steel fibres (2.355 kg/m^3^, +1.81%), due to the mass of steel fibres. This increases the overall mass of SCC, which may impact the load of the structure and transport. It can reduce the workability and increase the water absorption of concrete, requiring the optimisation of the mixture. Steel fibres are suitable for floors and structures exposed to high dynamic loads.

### 3.3. Assessment of the Research in the Context of CO_2_ Emissions and the Possibility of Their Reduction by Using Alternative Materials

Concrete production is one of the main sources of carbon dioxide (CO_2_) emissions, mainly due to the burning process of cement clinker, therefore any modification to the concrete composition, e.g., by using fibres or alternative materials, should consider the potential impact on emissions reduction. The use of polypropylene (WPO), glass (WSZ), and steel (WST) fibres in self-compacting concrete (SCC) can have both a positive and negative impacts on CO_2_ emissions.

Positive aspects:

- Increased durability—concretes with added fibres exhibit better resistance to bending, abrasion, and frost, which extends their service life and reduces the need for repairs, thereby lowering emissions associated with maintenance and reconstruction.
- Improved frost resistance (WSZ and WST)—enhanced performance during freeze–thaw cycles minimises structural damage, reducing the need for repairs and renovations, and the consumption of cement, which in turn helps lower emissions.
- Mix optimisation—the potential to reduce the amount of cement used while maintaining high mechanical performance (especially in SCC reinforced with WSZ) can significantly contribute to lowering CO_2_ emissions.

Negative aspects: Fibre production also generates CO_2_ emissions:

- Steel fibres (WSTs)—have the largest carbon footprint due to the energy consumption involved in their manufacture.
- Glass fibres (WSZs)—require high temperatures for production, which also generates emissions, though lower than those associated with steel fibres.
- Polypropylene fibres (PPFs)—are derived from petroleum, resulting in emissions related to the processing of petrochemical resources.

To reduce CO_2_ emissions in the production of SCC, the following solutions can be used:

- Reducing the amount of cement through the use of mineral additives.

Fly ash (PFA)—a by-product of coal combustion, can substitute 30–50% of cement, and reduce CO_2_ emissions by up to 40%. Blast furnace slag (GGBS)—a by-product of steelmaking, can replace 30–70% of cement, reducing CO_2_ emissions and improving the durability of concrete. Microsilica—increases the strength of concrete, allowing for a reduction in the amount of cement. Fibres can additionally improve the mechanical properties of concrete with these additives, which allows for further reduction of cement.

- Use of cements with lower CO_2_ emissions.

Belite cements (BCTs)—They contain less clinker, which reduces CO_2_ emissions by 30–40%. Geopolymer cements—An alternative based on the alkaline activation of ash and slag, can reduce emissions by up to 70–80%. Fibres can improve the resistance of geopolymer SCC to cracks and shrinkage, increasing its durability.

- Use of natural fibres instead of synthetic ones.

Basalt fibres—produced from volcanic lava, have a lower carbon footprint than glass and steel fibres. Hemp, flax, and cellulose fibres—biodegradable and environmentally friendly, but require additional protection against moisture. The use of natural fibres in SCC can be beneficial in lightweight structures and concrete with reduced mechanical load.

To reduce the carbon footprint of self-compacting concrete, it is necessary not only to modify its composition by adding fibres, but also to strive to reduce the amount of cement by using mineral additives and cements with lower CO_2_ emissions. Glass fibres (WSZs) seem to be the best choice, because they provide high strength and durability, while not generating as much CO_2_ emissions as steel fibres. The most ecological approach to SCC is a combination of glass or natural fibres, low-emission cements, and cement-replacing additives. This strategy can reduce CO_2_ emissions by up to 50–70% while maintaining the high durability of concrete.

## 4. Conclusions

The conducted study allows for the evaluation of the impact of different types of fibres (steel, glass, and polypropylene) on key properties of self-compacting concrete (SCC), both in its fresh and hardened states. Considering sustainable development requirements and the need to reduce CO_2_ emissions in the construction sector, the results indicate potential directions for the development of sustainable and durable concrete mixtures.

Detailed Conclusions:

- Compressive Strength—Glass fibres (GFs) improved compressive strength (+1.6%) and proved to be the most effective where concrete is required to exhibit high durability. The presence of steel (SF) and polypropylene fibres (PPFs) decreased compressive strength, which may be due to increased porosity or the suboptimal internal structure of the mix.
- Flexural Strength—The greatest increase in flexural strength was observed with glass fibres (+29.2%), suggesting their usefulness in structures exposed to dynamic loads and cracking risk. Steel fibres also significantly improved flexural strength (+18.8%), while polypropylene fibres, although to a lesser extent (+12.5%), still offered improvements over the reference mix.
- Water Absorption—The lowest water absorption was recorded in SCC with glass fibres (−10.9%), which translates into better durability in environments exposed to moisture and freeze–thaw cycles. Polypropylene fibres also reduced water absorption (−7.3%), which is important for elements prone to shrinkage and degradation. Steel fibres increased absorption (+26.6%), limiting their use in humid environments without additional protection.
- Density—The greatest increase in density was recorded in SCC with steel fibres (+1.81%) and polypropylene fibres (+1.77%), potentially increasing the self-weight of structural elements. The most optimal combination of density and strength was achieved with glass fibres (+0.99%), confirming their high performance.

General Conclusions:

1. Glass fibres (GFs) show the best overall impact on mechanical properties and durability of SCC. They can contribute to reducing the need for repairs and extending the service life of structures, indirectly supporting CO_2_ reduction goals. They are recommended for use in precast elements, load-bearing structures, and constructions exposed to dynamic loads.
2. Polypropylene fibres (PPFs) improve water absorption resistance and are particularly suitable for structures exposed to high humidity and shrinkage risks (e.g., tunnels, insulation, and fire-resistant components). Their low weight and thermal properties may support the design of lighter, more sustainable structures.
3. Steel fibres (SFs) enhance flexural strength, making them suitable for industrial floors and other elements subjected to heavy dynamic loads. However, their increased water absorption limits their use in aggressive environments without additional protective measures.
4. The use of fibres as additives to SCC can form part of a broader CO_2_ reduction strategy in construction—by increasing durability, reducing repair frequency, and partially replacing cement. While fibres themselves do not directly reduce emissions from cement production, their application aligns with broader sustainable development objectives.
5. This research results indicate that the optimal selection of fibre type and quantity can not only improve SCC properties but also increase its durability, thereby reducing the overall carbon footprint over the structure’s life cycle.

## Figures and Tables

**Table 1:** Physicochemical properties of CEM II/A-V 42.5 R cement [41].

Properties	Assumptions	Test Results
sulphate content (as SO_3_)	4.0	2.89
chloride content [%]	≤0.10	0.058
start of setting time [min]	≥60	214
end of setting time [min]	-	251
volume stability [mm]	≤10	1.5
water demand [%]	-	29.2
compressive strength [MPa] after 2 days/after 28 days	≥20.0	24.1
	≥42.5 and ≤62.5	52.4
specific surface area [cm^3^/g]	-	4620
alkali content Na_2_O_eq_ [%]	-	0.79

**Table 2:** Properties of the Isoplast 1528 [44] and Isoflow 7495 admixtures [45].

Properties	Isoplast 1528	Isoflow 7495
density [g/cm^3^]	1.14 ± 0.03	1.07 ± 0.02
form	liquid	liquid
color	brown	light yellow
pH	5.0 ± 1.0	4.0 ± 1.0
content Cl^−^ [%]	≤0.10%	≤0.10
content Na_2_O [%]	≤1.50%	≤1.50

**Table 3:** Composition of concrete mix per 1 m^3^.

Type of Concrete	Concrete-Mix Components [kg/m^3^]						
	Water	Sand	Gravel	Cement	Fibres	Isoplast	Isoflow
BZ	160	640	960	380	-	2.67	5.70
WPO	160	634	951	380	3.00	2.67	7.22
WSZ	160	634	951	380	5.00	2.67	6.84
WST	180	613	920	380	25.00	2.67	6.27

**Table 4:** Concrete-mix test results.

Type of Concrete	Properties of Concrete Mix				
	Consistency/Flow Diameter [mm]	Density [kg/m^3^]	Air Content [%]	Viscosity [s]	PL2 Throughput Rate
BZ	SF1/625	2.298	3.0	6.5/VF1	0.90
WPO	SF1/595	2.292	3.0	9.5/VF2	0.80
WSZ	SF1/600	2.296	3.0	6.5/VF1	1.00
WST	SF1/620	2.314	3.0	7.0/VF1	0.95

**Table 5:** Frost resistance test results.

Type of Concrete	Average Compressive Strength [MPa]		Average Strength Loss of Samples Subjected to Freezing [%]	Average Weight [g]		Average Weight Loss [%]
	Base Samples	After 150 Freezing Cycles		Before Freezing	After 150 Freezing Cycles	
BZ	63.89	36.08	43.53	2337.2	2342.8	0.242
WPO	60.95	39.62	35.00	2372.2	2401.3	1.226
WSZ	64.12	37.74	41.14	2333.7	2367.3	1.443
WST	60.96	37.46	38.55	2380.1	2410.5	1.278

## Data Availability

Data are available for request from corresponding authors.
